# Effective Management of a Skin and Soft Tissue Infection Caused by Community-Acquired MRSA Through Triple-Targeted Therapy Along with Aggressive Source Control: A Case Report

**DOI:** 10.3390/idr17020027

**Published:** 2025-03-24

**Authors:** Matteo Laratta, Stefano Agliardi, Matteo Sola, Stefano Spina, Roberto Fumagalli

**Affiliations:** 1 Department of Anaesthesia and Critical Care, ASST Grande Ospedale Metropolitano Niguarda, 20162 Milan, Italy; matteo.laratta@ospedaleniguarda.it; 2 Postgraduate School of Clinical Pharmacology and Toxicology, Department of Medical Biotechnology and Translational Medicine, University of Milan, 20122 Milan, Italy; stefano.agliardi@ospedaleniguarda.it; 3 Chemical-Clinical Analyses Unit, ASST Grande Ospedale Metropolitano Niguarda, 20162 Milan, Italy; 4 Post-Graduate School of Anesthesia, Intensive Care and Pain Medicine, School of Medicine and Surgery, University of Milano-Bicocca, 20126 Milan, Italy; m.sola5@campus.unimib.it; 5 Department of Anesthesia, Critical Care and Pain Medicine, Massachusetts General Hospital and Harvard Medical School, Boston, MA 02114, USA

**Keywords:** MRSA, skin and soft tissue infections, septic shock, source control, ECCO_2_-R, triple-targeted therapy

## Abstract

**Background:** Methicillin-resistant *Staphylococcus aureus* (MRSA) is a significant cause of healthcare-associated infections in Europe. It has become increasingly prevalent in community settings, causing skin and soft tissue infections (SSTIs). Managing community-acquired (CA) MRSA infections is challenging due to its high virulence and resistance to common antibiotics, and prevention outside the hospital setting is complex. Combination therapy has demonstrated efficacy in the treatment of severe MRSA infections. Furthermore, surgical source control is critical in treating CA-MRSA infections, involving removing the primary infection site to interrupt bacterial replication. Timeliness and a correct surgical approach are essential for successful treatment outcomes and improved quality of life. **Methods:** This report details the case of a 15-year-old athlete who was admitted to the intensive care unit with septic shock caused by CA-MRSA. **Results:** Despite initial treatment, his condition rapidly worsened. A computed tomography (CT) scan identified multiple abscesses (in the lungs, limbs, thyroid, and subscapular region) along with other complications. To achieve adequate tissue concentrations at all affected sites, a triple-targeted antimicrobial therapy was initiated and adjusted based on therapeutic drug monitoring (TDM). At the same time, daily surgical debridement was performed. The patient responded significantly to this treatment, and blood cultures eventually returned negative. **Conclusions:** A multidisciplinary approach involving early source control, tailored antimicrobial therapy, and, if monotherapy fails to control infection, combination therapy is advisable to treat life-threatening CA-MRSA infections.

## 1. Introduction

According to the European Center for Disease Prevention and Control (ECDC), methicillin-resistant *Staphylococcus aureus* (MRSA) represents 16.7% of *S. aureus* isolates [1]. MRSA is a leading cause of healthcare-associated infections (HAIs). ECDC estimates that among all HAIs caused by antibiotic-resistant bacteria in Europe, MRSA is responsible for nearly 44% of cases and over 20% of excess mortality [2]. Previously restricted to the hospital environment, this microorganism is now more widespread outside healthcare facilities [3,4]. Skin and soft tissue infections (SSTIs) caused by community-associated MRSA have been increasing over the last decade [5].

For this reason, a specific community-acquired MRSA (CA-MRSA) strain has been identified. CA-MRSA SSTIs can manifest in various clinical forms, such as abscesses, folliculitis, cellulitis, and toxic shock syndrome. Containing the spread of CA-MRSA in the community is extremely difficult [6]. Additionally, managing these infections is challenging, as this microorganism is very virulent and can resist many commonly used antibiotics.

The choice of the best antibiotic should also include the evaluation of multiple factors. More traditional options, such as vancomycin, benefit from proven efficacy and low costs but are burdened by limitations in terms of pharmacokinetics and side effects (especially renal toxicity). Widely used in the critical setting are drugs such as linezolid or daptomycin, with the first one presenting bacteriostatic activity and excellent pharmacokinetic/pharmacodynamic (PK/PD) characteristics but still associated with hematological toxicity and need for monitoring, and the second one being bactericidal but inadequate for the treatment of some specific foci, such as lung infections. New options like ceftaroline, ceftobiprole, or delafloxacin seem promising in terms of efficacy, pharmacokinetics, and tolerability but are burdened by higher costs and less availability of evidence. Finally, long-acting options such as dalbavancin or oritavancin may be promising, even if their use in the critical setting remains limited [7]. As an option, the most recent evidence highlights the effectiveness of combination therapy, compared to monotherapy, in managing severe MRSA infections [7,8]. Except for treating endocarditis, triple-targeted therapy is not usually used in treating MRSA infections.

Surgical source control is crucial for treating CA-MRSA infections, as it involves removing the primary site of infection, which is usually considered the main entrance door for the bacteria into the body, to interrupt the bacteria’s replication cycle [9,10]. Different surgical techniques are used depending on the type and extent of the infection, especially in the case of skin infections, incision and drainage might be enough. At the same time, more invasive surgical intervention may be required for internal organ or deep tissue infections, such as lung infections, osteomyelitis, or endocarditis [11]. The success of the surgery depends on its timeliness, which can improve treatment outcomes and quality of life [12].

In this report, we present the case of a young athlete who was admitted to our intensive care unit due to CA-MRSA sepsis, where the roles of triple-targeted therapy and source control were crucial for the management of the infection.

## 2. Case Description

**Figure 1:**
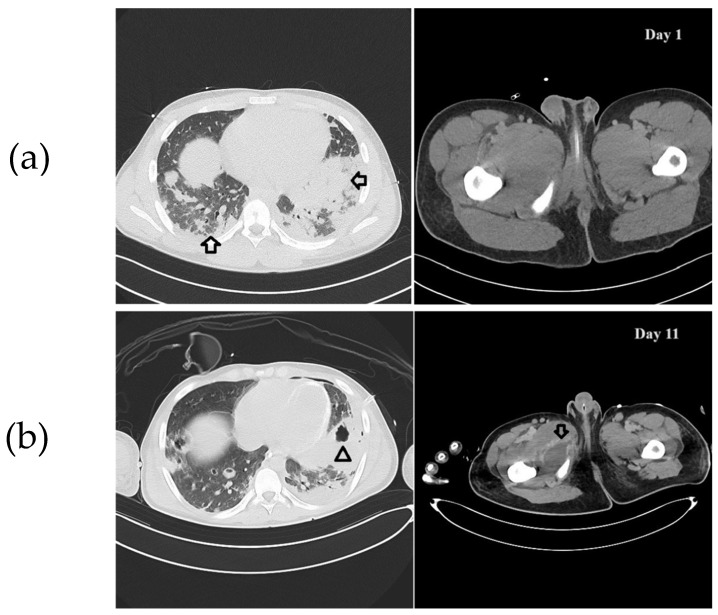
(**a**) CT scan imaging on day 1. On the left, chest CT. Arrows indicate the abscesses affecting the lung parenchyma. On the right, thigh CT. No abscess is evident. (**b**) CT scan imaging on day 11. On the left, chest CT. The arrowhead indicates cavitation of an abscess. On the right, thigh CT. The arrow indicates an abscess at the medial region of the thigh.

A 15-year-old boy of North African origins presented to the emergency department (ED) of Niguarda Hospital (Milan, Italy) with thigh pain, asthenia, and dyspnea after a minor trauma occurred during a soccer game. On assessment, he was hypoxic, hypotensive, and tachycardic. On blood tests, he had increased hepatolysis indices (Aspartate aminotransferase/Alanine transaminase 325/133 U/L), creatinine values (1.7 mg/dL), C-Reactive protein (31.8 mg/dL), procalcitonin values (59 ng/mL), troponin (75 ng/L), and pro-BNP (3792 ng/L) Three days earlier, he had already been evaluated in another ED for thigh pain bilaterally and treated with a non-steroidal anti-inflammatory drug with only transient benefits. His past medical history was unremarkable. Circulatory shock with hyperlactatemia and severe hypoxemic respiratory failure rapidly arose. A chest X-ray was performed and showed thickening of the left basal lung and increased interstitial pattern with costophrenic sinus obliteration. An echocardiogram was then conducted, which showed mild septal dyskinesia without severe valvular dysfunction or pericardial effusion. Given the deterioration of the clinical condition and the hemodynamic instability, in order to manage respiratory failure, set the ventilatory parameters, and diagnose any complications, a whole-body computed tomography (CT) scan showed a lung pattern with ground glass and diffuse consolidations (Figure 1a, on the left). Furthermore, hepatosplenomegaly, diffuse lymphadenopathy, homolateral femoral vein thrombosis without pulmonary embolism, and an inhomogeneous appearance of the thyroid gland were diagnosed. No significant alterations were found at the level of the painful thigh (Figure 1a, on the right). The SARS-CoV-2 screening swab was negative. Severe respiratory failure occurred (respiratory rate 50/min, PaO_2_/FiO_2_ ratio 220 mmHg). Orotracheal intubation was performed, and mechanical ventilation was initiated. Hemodynamic support with high-dose norepinephrine and vasopressin was started (norepinephrine 0.67 mcg/kg/min and vasopressin 0.013 U/min). Blood cultures were drawn, and an empirical antimicrobial therapy was started with vancomycin and piperacillin/tazobactam. Since the co-existence of a lymphoproliferative disease could not be excluded, empirical antifungal therapy (then discontinued within 24 h) with liposomal amphotericin B was also started. A lumbar puncture was performed, but the cerebrospinal fluid was not suggestive of meningitis. Given the lymphadenomegaly and hepatosplenomegaly, elevated LDH, and elevated IL-6, a lymphoproliferative disease was hypothesized, and a lymph node biopsy was performed, which reported a predominantly reactive pattern. When macrophage activation syndrome (MAS) was suspected, the clinical severity did not allow a bone marrow biopsy to be performed, but the peripheral blood smear and the presence of positive blood cultures for MRSA were consistent with septic shock, abandoning the hypothesis of macrophage activation syndrome.

When blood cultures were positive for MRSA, empirical antimicrobial therapy was suspended, and a narrow-spectrum therapy with linezolid and ceftaroline was started. Bronchoalveolar lavage and multiple tissue biopsies were also positive for MRSA. Panton–Valentine Leucocidin was not present. Pediatric surgeons were asked to evaluate the sore thigh, but neither radiologic findings nor clinical examination highlighted a suspected lesion that needed to be drained.

However, in the next few days, the clinical conditions did not improve. Bilateral pneumothorax occurred, and ultra-protective mechanical ventilation was instituted with extracorporeal CO_2_ removal (ECCO_2_-R). The start of ECCO_2_-R (blood flow 400 mL/min, fresh gas flow 15 L/min) allowed us to reduce driving pressure from 17 cmH_2_O to 13 cmH_2_O and minute ventilation from 11.5 L/min to 6 L/min. The impact on oxygenation was modest (PaO_2_/FiO_2_ ratio from 190 to 150 mmHg). Continuous renal replacement therapy (CRRT) was initiated for severe acute renal injury. Refractory fever was present despite extracorporeal circuits. Multiple serial blood cultures from all catheters placed on patients and from an extemporaneous peripheral venous sample were positive in at least two specimens from different sites for more than one week. Although the difference in time to positivity between peripheral and central venous catheters was always less than two hours, it was decided to replace the catheters seven days apart to exclude any residual suspicion of catheter-related bloodstream infection, but blood cultures sent subsequently were still positive. The infectious disease specialist recommended the implementation of daptomycin as a third-line targeted antibiotic therapy, given the patient’s ongoing critical condition. Serial therapeutic drug monitoring (TDM) for linezolid was performed, and continuous infusion was adjusted to 1800 mg/day. 

Given the persistence of bacteriemia ten days after admission, it was decided to reassess the patient because of the suspicion of misdiagnosed septic foci. The multi-organ failure requiring high amine doses, CRRT, and ECCO_2_r did not allow for time-consuming methods such as MRI or long transports to the nuclear medicine department for a PET/CT scan, so it was decided to perform a whole-body CT scan to re-examine the lungs, but also to search for possible occult septic foci in the limbs and soft tissues. This new exam showed multiple abscess collections (lungs, limbs, and subscapular region). Thus, extensive surgical debridement was performed (Figure 1b). Notably, a thyroid abscess was also drained. The source control was achieved after a wide soft tissue debridement and serial Vacuum-Assisted Closure (VAC) therapies.

**Figure 2:**
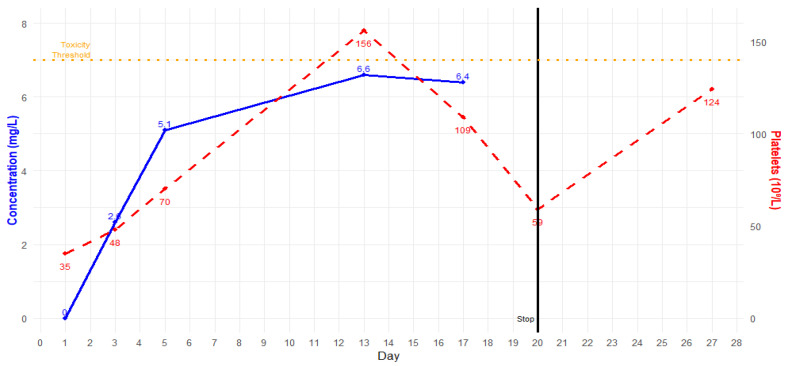
Linezolid trough plasma concentration (blue) and platelet count (red) in time.

**Figure 3:**
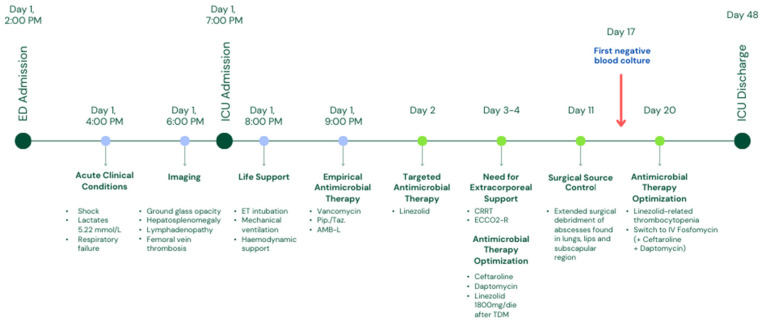
Case timeline. Abbreviations: ED = emergency department, ICU = intensive care unit, ET = endo-tracheal, Pip./Taz. = piperacillin/tazobactam, AMB-L = liposomal amphotericin B, CRRT = continuous renal replacement therapy, ECCO_2_-R = extracorporeal CO_2_ removal, TDM = therapeutic drug monitoring, IV = intravenous.

After two weeks of antibiotic therapy, thrombocytopenia occurred (Figure 2). As per the infectious disease specialist’s recommendation, linezolid was substituted with intravenous fosfomycin (after in vitro susceptibility confirmation). Fifteen days after hospitalization, blood cultures resulted negative, and no other infections were detected. Respiratory and renal function progressively improved, and ECCO_2_-R and CRRT were discontinued after 24 days of treatment. The patient progressively improved, and complete respiratory and hemodynamic weaning was achieved. The patient was extubated after 30 days. Antibiotic therapy was continued for 12 weeks, and no other complications occurred. Figure 3 and Table 1 summarize the timeline of this case.

## 3. Discussion

In this case report, we present the management of a diffuse SSTI caused by CA-MRSA complicated by septic shock and MOF in a young 15-year-old boy athlete. Among the multiple secondary foci, the finding of thyroid abscess was very peculiar. Antibiotic combination therapy and aggressive source control were cornerstones in the management of such a severe case. Some points in the management of this case are worth mentioning.

First, blood cultures were persistently positive for over a week despite a triple-targeted therapy (with a linezolid plasmatic concentration in the therapeutical range). Infections caused by drug-resistant Gram-positive cocci are classically managed with single-drug therapies. According to the 2011 Clinical Practice Guidelines issued by the Infectious Diseases Society of America for the Treatment of Methicillin-resistant *Staphylococcus aureus* Infections in Adults and Children [13], cases of MRSA bloodstream infections that a single antibacterial agent does not effectively control should be addressed with drug combinations.

Linezolid was introduced as a first-line therapy to treat MRSA infections, as per our institution guidelines, which are based on the protocols developed by the working groups of the Italian intensive care society [7]. Linezolid has excellent efficacy, broad spectrum of activity against Gram-positive bacteria, relative lack of resistance, as well as favorable pharmacokinetics, which allow for high penetration into most tissues, including lungs and soft tissues, but also within intra-abscess areas [14]. Some of the most prominent reference guidelines, such as the IDSA guidelines [13], which represent a significant milestone in the treatment of MRSA infections in both adult and pediatric patients, support the use of linezolid as a potential first-line agent for MRSA pneumonia. These guidelines assign it a strength of recommendation and quality of evidence class AI/II (adults/pediatrics) comparable to that of vancomycin. Moreover, evidence suggests a higher clinical cure rate and better microbiological clearance in MRSA pneumonia patients treated with linezolid compared to vancomycin. Additionally, linezolid demonstrates more favorable characteristics, such as reversibility of potential toxicity and superior pharmacokinetics, particularly in terms of penetration into ELF (epithelial lining fluid) and abscesses [15]. These features make it the antibiotic of choice for managing this type of infection in routine clinical practice at our institutions. Furthermore, given the inability to initially rule out PVL-toxin production by the bacterium, we believe the use of a protein synthesis inhibitor like linezolid was the most appropriate therapeutic choice. The same applies to SSTIs, where linezolid is recommended with a strength of recommendation and quality of evidence rating of AI/AII, comparable to that of vancomycin.

Daptomycin is unsuitable in the treatment of pneumonia; however, it is known for its rapid bactericidal effect on MRSA and optimal soft tissue penetration, representing a potentially ideal treatment choice for SSTIs. The same IDSA guidelines place it among the antibiotics of choice in case of SSTIs, with a strength of recommendation and quality of evidence class AI (same as vancomycin and linezolid) among adults and as a promising agent among pediatric patients. Numerous in vitro and in vivo studies and case reports have demonstrated the efficacy of combining daptomycin with linezolid, considering the distinct pharmacodynamic targets of these two drugs. When used together, linezolid significantly augments the bactericidal effect of daptomycin, effectively combating both planktonic bacteria (PB) and biofilm-embedded bacteria (BB) within 48 h. Consequently, combination therapy involving daptomycin and linezolid may prove more effective than monotherapy [16].

Ceftaroline is emerging as a promising bactericidal agent against MRSA infections (including pneumonia), exhibiting favorable efficacy and pharmacokinetic/pharmacodynamic (PK/PD) characteristics (including low protein binding), ensuring robust penetration into the epithelial lining fluid (ELF), soft tissues, and abscesses. Ceftaroline demonstrated good efficacy in managing complicated infections, due to its bactericidal action and wide tissue penetration [17]. Moreover, the ceftaroline’s hallmark is represented by a specific mechanism, targeting MRSA PBP2a with high affinity, thereby ensuring an additive effect when combined with other agents such as linezolid and daptomycin [18].

In this case, characterized by a critical, multisite infection supported by a difficult-to-treat bacterium such as MRSA, the strategy was to ensure maximum coverage, seeking both a pharmacodynamic synergism between antimicrobials, while including molecules with broad tissue distribution. In this perspective, the strategy was to combine at least two synergistic agents with favorable PK/PD profiles in each of the infected tissues: linezolid–ceftaroline for pneumonia and linezolid–daptomycin (and also ceftaroline) for soft tissues and musculoskeletal foci.

TDM for linezolid is crucial for correct dosage titration, especially among critically ill patients, due to the high variability and complex management of these subjects [15,16]. In particular, critically ill septic patients with acute kidney injury (AKI) who are undergoing CRRT could experience significant changes in linezolid PK/PD parameters [19,20]. In this case, we had to increase the linezolid dosage to 1800 mg/day to reach the target (C_min_ 2–7 mg/L), as widely suggested in the literature [21,22]. Despite adequate linezolid dosing after adjustment, the patient suffered from progressive thrombocytopenia after two weeks of treatment. Therefore, we replaced linezolid with intravenous fosfomycin. The decision to replace linezolid with fosfomycin is supported by evidence of its synergistic effect with both daptomycin and ceftaroline. While daptomycin and ceftaroline disrupt *S. aureus* cell membrane synthesis, fosfomycin complements this action by inhibiting the early stages of peptidoglycan synthesis, enhancing the efficacy of both these antibiotics [23]. Moreover, thanks to its low molecular weight (138.06 g/mol) and protein binding, fosfomycin is known for its excellent penetration into the lungs, muscles, and abscesses, making it a suitable choice for a case like the one described [24,25]. Triple anti-MRSA therapy proved to be safe, and no other drug-related adverse effects were reported. The triple-targeted therapy was continued for 12 weeks.

Although the literature is not exhaustive in declaring the superiority of combination therapy over antimicrobial monotherapy, some authors agree on the difficulty in treating critical cases such as this one, where the fragility of the patient, the presence of multi-resistant organisms, the multiplicity and different localization of infectious foci, and the difficulty in performing rapid and effective source control could compromise the efficacy of antimicrobial therapy, especially in case of TDM unavailability [26]. Regarding adverse events (AEs), the use of a greater number of agents suggests more careful monitoring of toxicities, even if in an ICU setting where patients are closely monitored (e.g., CPR and eosinophilia for patients receiving daptomycin, and lactate levels and platelet counts for those on linezolid, hypernatremia for fosfomycin). The benefits of an intensified antibiotic regimen may significantly outweigh the risks, especially in centers equipped with clinical pharmacology and therapeutic drug monitoring services.

Second, soon after ICU admission, pediatric surgeons evaluated the patient for an early surgical debridement, but no evident focus was identified. Furthermore, endocarditis was ruled out through serial echocardiographic exams. Patients with a positive follow-up blood culture should undergo a thorough evaluation for occult foci of infection and an immediate surgical debridement [27]. Only a high index of suspicion and the presence of a multidisciplinary team made it possible to identify metastatic foci as soon as they were evident. Source control is a cornerstone of sepsis treatment and should be performed as early as possible to reduce mortality [28]. After effective source control, blood cultures resulted negative within a few days, and clinical conditions improved. Therefore, surgical debridement was necessary to obtain an infection cure, regardless of the correct antibiotic therapy. This case showed that source control is crucial, especially in persistent bacteremia, even in cases where the primitive focus appears uncertain or small. VAC therapy can be a helpful tool in managing extensive tissue debridement.

Lastly, ECCO_2_-R can be valuable in ensuring ultra-protective mechanical ventilation in the presence of multiple lung abscesses [29,30]. The initiation of treatment was carried out without anticoagulation. After the patient’s stabilization, anticoagulation was maintained with unfractionated heparin at a dose of 800–1400 U/h.

## 4. Conclusions

MRSA may cause life-threatening diffuse infections among pediatric and young adult healthy patients. A multidisciplinary approach, characterized by source control, effective combination therapy tailored through fast microbiology and TDM, and advanced management of organ complications, is needed to combat these severe infections adequately. Microbiologists, infectious disease specialists, clinical pharmacologists, and surgeons should be involved to ensure optimal support to intensivists and provide a 360-degree view of such complex cases.

## Figures and Tables

**Table 1:** Laboratory test panel referring to the time-points reported in Figure 3. NA = not available.

Parameter	ED Admission (2:00 PM)	ED T1 (4:00–6:00 PM)	ICU Admission (7:00 PM)	ICU Day 1	ICU Day 2	ICU Day 4	ICU Day 11	ICU Day 17	ICU Day 21	ICU Discharge
WBC (10^9^/L)	4.56	6.45	6.05	12.6	21.96	24.4	21.5	9.86	9.05	8.2
Hb (g/dL)	13.7	15	13.4	9.8	9.5	9.6	7.8	8	9.4	8.7
HCT (%)	40.9	45.4	41.6	30.9	29.4	30	24.2	24.5	29.9	27.4
PLT (10^9^/L)	37	49	34	48	61	70	203	130	59	140
INR	1.37	1.27	1.37	1.57	1.39	1.32	1.02	0.98	1.02	1.25
PTT ratio	1.77	1.57	1.65	1.61	1.29	1.07	1.1	1.73	1.17	1.16
Mioglobin (ug/L)	NA	NA	3374	5312	1341	422	1277	288	384	26
CK-MB (ng/mL)	NA	19.6	23.1	68.5	33.4	4.8	6.8	3.1	4.5	2
T-Troponine (mg/dL)	75	877.4	1046	555	357	860	248	73	99.4	101.1
CK (U/L)	2136	3103	2039	4106	2753	592	72	95	91	13
Lactate (mmol/L)	4.56	5.22	4.64	3.22	2.65	1.65	2.42	2.46	1.88	1
Creatinine (mg/dL)	1.57	1.73	1.5	1.68	1.18	0.79	1.22	0.37	0.84	1.02
Urea (mg/dL)	115	108	122	132	113	100	89	51	36	47
Na^+^ (mmol/L)	131	128	134	134	135	140	130	136	138	136
K^+^ (mmol/L)	3.8	3.8	3.9	4.5	4.6	4	4.1	4.3	3.3	3.8
Albumin (g/dL)	NA	NA	2.68	3.05	2.56	2.78	1.94	3.21	2.62	2.66
AST (U/L)	NA	325	268	206	153	89	76	265	53	30
ALT (U/L)	109	133	110	86	93	80	49	185	79	21
Bilirubin tot. (mg/dL)	4.17	4.85	4.59	4.06	3.66	6.36	7.6	8.96	1.67	0.78
Pro-BNP (ng/L)	5601	NA	7949	14,239	14,124	7419	5118	701	6670	15,066
LDH (U/L)	729	NA	572	627	588	543	375	227	207	232
Ferritine (ng/mL)	2965	NA	1876	NA	NA	1632	NA	NA	NA	747
Procalcitonin (ng/mL)	83.18	NA	80.55	NA	48.64	22.6	3.66	2.09	1.54	0.27
CRP (mg/dL)	31.8	NA	NA	NA	NA	NA	NA	NA	NA	NA

## Data Availability

The authors are available for contact regarding any further information of interest related to the case.
